# Lung IFNAR1^hi^ TNFR2^+^ cDC2 promotes lung regulatory T cells induction and maintains lung mucosal tolerance at steady state

**DOI:** 10.1038/s41385-020-0254-1

**Published:** 2020-01-20

**Authors:** Samira Mansouri, Divya S. Katikaneni, Himanshu Gogoi, Mauricio Pipkin, Tiago N. Machuca, Amir M. Emtiazjoo, Lei Jin

**Affiliations:** 1 Division of Pulmonary, Critical Care and Sleep Medicine, Department of Medicine, University of Florida, Gainesville, FL 32610 USA; 2 Division of Thoracic and Cardiovascular Surgery, Department of Surgery, University of Florida, Gainesville, FL 32610 USA

## Abstract

The lung is a naturally tolerogenic organ. Lung regulatory T cells (T-regs) control lung mucosal tolerance. Here, we identified a lung IFNAR1^hi^TNFR2^+^ conventional DC2 (iR2D2) population that induces T-regs in the lung at steady state. Using conditional knockout mice, adoptive cell transfer, receptor blocking antibodies, and TNFR2 agonist, we showed that iR2D2 is a lung microenvironment-adapted dendritic cell population whose residence depends on the constitutive TNFR2 signaling. IFNβ-IFNAR1 signaling in iR2D2 is necessary and sufficient for T-regs induction in the lung. The Epcam^+^CD45^−^ epithelial cells are the sole lung IFNβ producer at the steady state. Surprisingly, iR2D2 is plastic. In a house dust mite model of asthma, iR2D2 generates lung T_H_2 responses. Last, healthy human lungs have a phenotypically similar tolerogenic iR2D2 population, which became pathogenic in lung disease patients. Our findings elucidate lung epithelial cells IFNβ-iR2D2-T-regs axis in controlling lung mucosal tolerance and provide new strategies for therapeutic interventions.

## Introduction

Lungs are constantly exposed to a variety of particles, allergens, and airborne microbes. Remarkably, this nonstop exposure generally results in tolerance instead of inflammation. Lung dendritic cells (DCs) are key inducers of lung tolerance.^1–4^ The induction of peripheral tolerance by DCs at the steady state is an active process that promotes the generation of peripheral regulatory T cells (pT-regs).^5^ Mice lack of pT-regs spontaneously developed T_H_2 pathologies at mucosal sites, e.g., allergic inflammation and asthma.^6^ The induction of lung T-regs can reverse asthma in mice.^7,8^

Lung DCs consist of functionally distinct subsets: the CD103^+^ conventional DC (cDC1), the CD11b^+^CD24^+^CD64^−^ conventional DC (cDC2), monocyte-derived CD11b^+^CD24^−^CD64^+^ DC (moDCs), and B220^+^SiglecH^+^CD11C^low^ plasmacytoid DCs (pDCs).^9^ cDC2 itself is a heterogeneous population, including a subpopulation of Klf4^+^/Mgl2^+^ cells promoting T_H_2 responses.^10–13^ cDC1 and pDCs were reported to induce T-regs in the lung.^2,14^ Lung Siglec F^+^ macrophage can also induce T-regs.^15^ Thus, it remains unclear which lung antigen-presenting cells (APCs) induce the lung T-regs at steady state.

Lung DCs also promote immunogenic responses. Lung cDC1 promotes the antigen cross-presentation and the induction of cytotoxic T lymphocyte responses. Lung cDC2 require IRF4 expression for development and have been shown to mediate house dust mite (HDM)-induced asthma.^10,13,16,17,18^ cDC2 also induces T_H_17,^19,20^ T follicular helper (T_FH_) responses.^12,21^ Noteworthy, the role of lung DCs to actively maintain lung tolerance at stead-state is exactly the opposite of the immunogenic roles during inflammation. It is unknown if there is a specific tolerogenic lung DC population or the same lung DC population promotes tolerogenic or immunogenic responses depending on the environmental cues.

Tolerogenic DCs induce T-regs by the expression of immunomodulatory molecules PD-L1/PD-L2, ICOS-L, and ILT3/4, and the production of immunosuppressive factors IL-10, TGFβ1, retinoic acid, and indoleamine 2,3-dioxygenase (IDO-1).^22^ Among them, TGFβ1 likely plays a central role in DCs-induced long-term peripheral tolerance.^23–25^ TGF-β1 promotes the conversion of peripheral naive T cells to T-regs.^23–25^ Modanelli, G. et al.,^26^ showed that TGFβ1-treated splenic DCs co-express IDO-1, arginase-1, and conferred long-term, immunosuppressive effects, which is essential for maintaining peripheral tolerance.^27^ Whether this IDO-1^+^Arg-1^+^ TGFβ-1-producing DCs population exists in vivo, such as in the tolerogenic lung, is unknown.

Here, we sought to identify the lung tolerogenic DC population and its underlying mechanism that induces lung T-regs at steady state. Unexpectedly, we revealed the plasticity of lung DCs.

## Results

### Lung TNFR2^+^ cDC2 population maintains lung mucosal tolerance at steady state

**Fig. 1:**
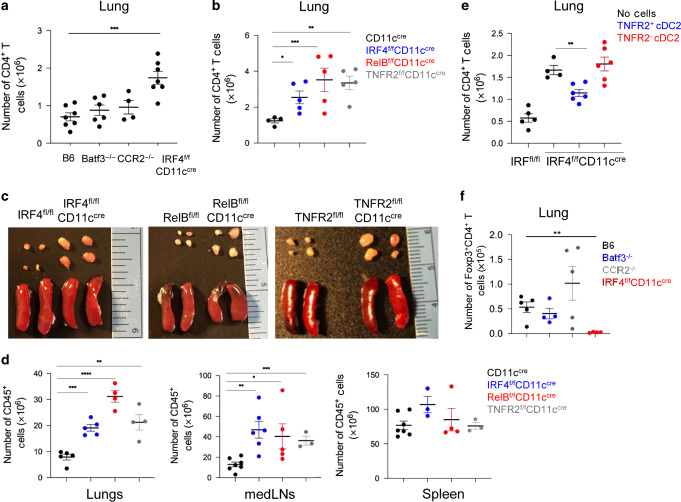
The lung TNFR2^+^ cDC2 population maintains lung tolerance and prevents lung inflammation at steady state. **a** Numbers of lung CD4^+^ T cells at steady state in C57BL/6 J (*n* = 7), Batf3^−/−^ (*n* = 6), CCR2^−/−^ (*n* = 4), and IRF4^fl/fl^CD11c^cre^ (*n* = 7) mice. Data were compiled from two independent experiments. **b** Numbers of lung CD4^+^ T cells in WT (*n* = 12), IRF4^fl/fl^CD11c^cre^ (*n* = 10), RelB^fl/fl^CD11c^cre^ (*n* = 7), and TNFR2^fl/fl^CD11c^cre^ (*n* = 5) mice at steady state. Data were representative of two independent experiments. **c** Image of mediastinal lymph nodes (mLNs; top) and spleens (bottom) in of 7- to 8-week-old knockout strains at steady state; *n* = 3 mice/group. Data are representative of two independent experiments. **d** Numbers of CD45^+^ cells in the lungs (left), mLNs (center), and spleens (right) in the indicated strains at steady state; *n* = 3 mice/group. Data were representative of two independent experiments. **e** Numbers of CD4^+^ T cells in IRF4^fl/fl^CD11c^cre^ mice 14 days post cell adoptive transfer. TNFR2^+^ or TNFR2^−^ cDC2 were sorted from WT mice lung and intranasally (i.n.) transferred into IRF4^fl/fl^CD11c^cre^ recipient mice; *n* = 4–6 mice/group. Data are representative of two independent experiments. **f** Numbers of lung Foxp3^+^CD4^+^ T-reg cells at steady state in C57BL/6 J, Batf3^−/−^, CCR2^−/−^, and IRF4^fl/fl^CD11c^cre^ mice; *n* = 4–5 mice/group. Data were representative of two independent experiments. Graphs represent the mean with error bars indication s.e.m. *P* values determined by one-way ANOVA Tukey’s multiple comparison test. *P < 0.05, **P < 0.001, and ***P < 0.0001.

We reasoned that lung mucosal tolerance is actively maintained by a specialized lung DC population, and mice lacking this tolerogenic lung DC population will spontaneously lose lung mucosal tolerance. We first examined lung CD4^+^ T cells in mice lacking different DC subsets, Batf3^−/−^ (cDC1), IRF4^fl/fl^CD11c^cre^ (cDC2), and CCR2^−/−^ (moDCs). Only the IRF4^fl/fl^CD11c^cre^ mice had spontaneously increased CD4^+^ T cells in the lung (Fig. 1a). Furthermore, IRF4^fl/fl^CD11c^cre^ mice had enlarged mediastinal lymph nodes (medLNs), but relatively normal spleens (Fig. 1c, d) suggesting a selective loss of lung tolerance by the lack of cDC2.

cDC2 is a heterogeneous population.^13,21^ We reasoned that to actively maintain lung tolerance, the tolerogenic cDC2 subset may be constitutively activated. A subpopulation of lung cDC2, marked by expression of TNFR2, has constitutively activated RelB (pRelB; Supplementary Fig. 1a, b).^21^ Notably, the TNFR2^+^ cDC2 (R2D2) population is the only lung DC subset that has constitutively activated RelB (Supplementary Fig. 1c). TNFR2^−/−^ mice lack the pRelB^+^ cDC2 subpopulation.^21^ Furthermore, the R2D2 population expresses tolerogenic DC markers of PD-L1, PD-L2, Arg-1, and BTLA.^21^ Last, the lack of RelB expression in DCs promotes the development of spontaneous allergic airway inflammation.^28^ We thus generated RelB^fl/fl^CD11c^cre^ and TNFR2^fl/fl^CD11c^cre^ mice to examine their lung mucosal tolerance at steady state.

Similar to the IRF4^fl/fl^CD11c^cre^ mice, RelB^fl/fl^CD11c^cre^ and TNFR2^fl/fl^CD11c^cre^ mice had increased immune cells in the lung and medLNs, but not the spleen (Fig. 1b–d). The IRF4^fl/fl^CD11c^cre^, RelB^fl/fl^CD11c^cre^, and TNFR2^fl/fl^CD11c^cre^ mice may delete IRF4, RelB, and TNFR2 in other lung CD11c^+^ cells. We examined RelB, IRF4, and TNFR2 expression in these mice. We found that while RelB^fl/fl^CD11c^cre^ deleted RelB in alveolar macrophage (AM), IRF4, and TNFR2 expression in lung AM were intact in the IRF4^fl/fl^CD11c^cre^ or TNFR2^fl/fl^CD11c^cre^ mice (Supplementary Fig. 1d–f). Nevertheless, to exclude the possibility that the lack of IRF4, RelB, or TNFR2 in lung cells other than R2D2 cause spontaneous lung inflammation, we did the adoptive cell transfer experiment. We sorted out lung TNFR2^+^ (R2D2) and TNFR2^−^ cDC2 from C57BL/6 mice and adoptively transfer intranasally (i.n.) them into the IRF4^fl/fl^CD11c^cre^ recipient mice. The quality of the adoptive cell transfer was confirmed after 24 h (Supplementary Fig. 1g–i). After 2 weeks, IRF4^fl/fl^CD11c^cre^ mice receiving wild-type (WT) R2D2 cells had their lung CD4^+^ T cells numbers reduced, while the IRF4^fl/fl^CD11C^cre^ mice receiving the WT TNFR2^−^ cDC2 still had elevated numbers of lung CD4^+^ T cells (Fig. 1e). Together, these data strongly suggested that lung R2D2 population maintains lung mucosal tolerance at steady state.

### Lung Mgl2^+^/IDO-1^+^ R2D2 population promotes lung T-regs induction at steady state

DCs promote tolerance via the generation of T-regs.^1–4^ We found that lung from IRF4^fl/fl^CD11c^cre^ had decreased T-regs at steady state (Fig. 1f). We reasoned that R2D2 cells induce T-regs in the lung. Intranasal administration of one dose of 1 µg innocuous protein antigens (e.g., OVA, PspA, H7N7-HA, and H1N1-NP) induces lung T-regs (Supplementary Fig. 2a). These lung T-regs were specific for the administered OVA antigen and neuropilin-1-negative pT-regs^29,30^ (Supplementary Fig. 2b, c). They also produce IL-10 (Supplementary Fig. 2d). Furthermore, intranasal administration of OVA turned adoptive transferred (i.v.) naive CD45.1^+^ T cells into Foxp3^+^ T-regs in vivo (Supplementary Fig. 1e). Last, consistent with the previous report,^3^ blocking DC migration by anti-CCR7 monoclonal antibody (mAb) inhibited OVA-induced lung T-regs induction (Supplementary Fig. 1f).

**Fig. 2:**
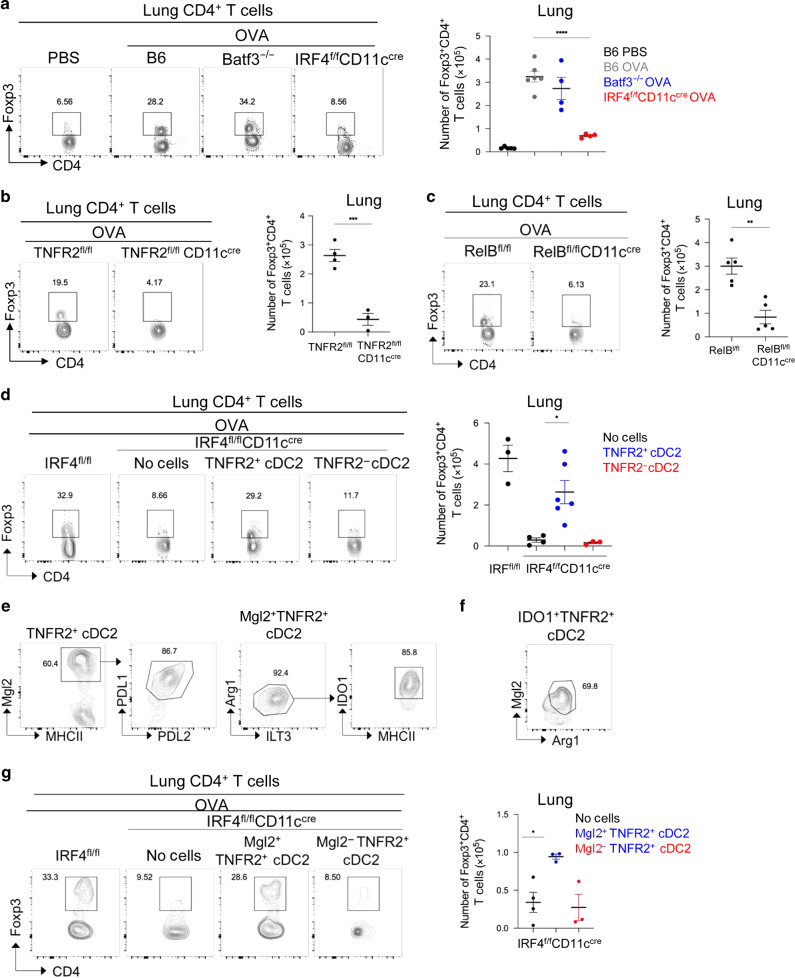
Lung-resident Mgl2^+^/IDO1^+^ R2D2 population generates T-regs in the lung. **a** Flow cytometry plots (left) and quantification (right) of CD4^+^Foxp3^+^ T-reg cells in in WT, Batf3^−/−^, and IRF4^fl/fl^CD11c^cre^ mice treated with one dose of OVA (1 μg) intranasally (i.n.). Lungs were harvested on day 14; *n* = 4–6 mice/group. Data are representative of two independent experiments. **b**, **c** Flow cytometry plots (left) and quantification (right) of T-regs in TNFR2^fl/fl^CD11c^cre^ **b** and RelB^fl/fl^CD11c^cre^ mice **c** treated with one dose of OVA (1 μg) i.n. Lungs were harvested on day 14; *n* = 3–5 mice/group. Data are representative of two independent experiments. **d** IRF4^fl/fl^CD11c^cre^ mice were adoptively transferred (i.n.) with lung TNFR2^+^ and TNFR2^−^ cDC2 from WT mice lung and treated with one dose of OVA (1 μg) i.n. Flow cytometry analysis (left) and quantification (right) of T-regs at day 14; *n* = 3–6 mice/group. Data are representative of two independent experiments. **e**, **f** Flow cytometry analysis of TNFR2^+^ cDC2 at steady state. Data are representative of three independent experiments; *n* = 3 mice/group. **g** IRF4^fl/fl^CD11c^cre^ mice were adoptively transferred (i.n.) with lung Mgl2^+^TNFR2^+^ and Mgl2^−^TNFR2^+^ cDC2 and treated with one dose of OVA (1 μg) i.n. Flow cytometry analysis (left) and quantification (right) of T-regs at day 14; *n* = 3–4 mice/group. Data are representative of two independent experiments. Graphs represent the mean with error bars indication s.e.m. *P* values determined by one-way ANOVA Tukey’s multiple comparison test **a**, **d**, **g** or unpaired student *t*-test **b**, **c**. *P < 0.05, **P < 0.001, and ***P < 0.0001.

The induction of lung T-regs by OVA depended on IRF4, RelB, or TNFR2 expression in CD11c^+^ cells but not Batf3, TNFR1, or CCR2 (Fig. 2a–c, Supplementary Fig. 2g). TNF2^fl/fl^LysM^cre^ mice, which delete TNFR2 in macrophage but not DCs (Supplementary Fig. 2h), had unaltered lung T-regs induction (Supplementary Fig. 2i). Last, adoptive transfer of R2D2 cells, but not lung TNFR2^−^ cDC2 increased lung T-regs induction in the IRF4^fl/fl^CD11c^cre^ mice (Fig. 2d). Notably, the rescue was incomplete and the likely due to technical difficulties (Supplementary Fig. 1i or other unknown contributors.

R2D2 cells express tolerogenic DC markers of PD-L1 and Arg-1.^21^ Closer examination found that a subpopulation of R2D2 expresses Mgl2 (Fig. 2e). The Mgl2^+^ R2D2 population is PD-L1^+^PD-L2^+^ILT3^+^Arg-1^+^ and constitutively express IDO-1 (Fig. 2e). Notably, all the IDO-1^+^ R2D2 cells are Mgl2^+^ and Arg1^+^ (Fig. 2f). The Mgl2^+^ DC population has been characterized before in the skin to promote T_H_2 responses or suppress T_FH_ response.^11,31^ We then adoptively transferred (i.n.) lung Mgl2^+^R2D2 and Mgl2^−^R2D2 cells into the IRF4^fl/fl^CD11c^cre^ mice and examined their ability to induce lung T-regs. Only Mgl2^+^R2D2 generated lung T-regs in the IRF4^fl/fl^CD11c^cre^ mice (Fig. 2g). Taken together, the lung Mgl2^+^/IDO-1^+^ R2D2 population induces lung T-regs at steady state.

### Constitutive TNFR2 signaling is required for the presence of R2D2 population in the lung at steady state

**Fig. 3:**
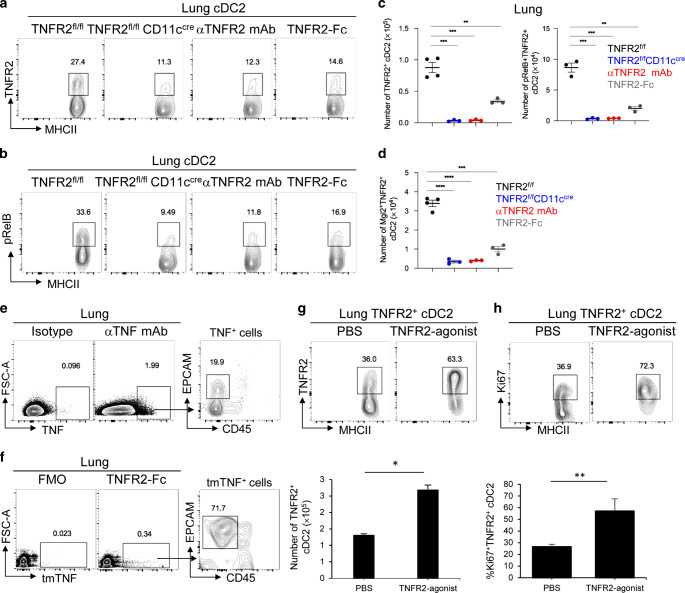
Tonic TNFR2 signaling is required for the presence of the R2D2 population in the lung. **a**–**c** Flow cytometry analysis of TNFR2 **a** and pRelB expression **b** on lung cDC2 in TNFR2^fl/fl^ and TNFR2^fl/fl^CD11c^cre^ mice at steady state. WT mice were treated i.n. with anti-TNFR2 blocking antibody (20 μg) or TNFR2-Fc recombinant protein (2 μg). Lungs were harvested 24 h later; *n* = 3–4 mice/group. Data are representative of two independent experiments. **d** Quantification of Mgl2^+^TNFR2^+^ cDC2 in the lungs of indicated groups; *n* = 3–4 mice/group. Data are representative of two independent experiments. **e**, **f** Flow cytometry analysis of TNF expression by anti-TNF mAb (clone D2D4) **e** or mouse TNFR2-Fc recombinant protein **f**; *n* = 3 mice/group. Data are representative of three independent experiments. **g**–**h** Flow cytometry analysis of TNFR2^+^ cDC2 **g** and Ki67 expression **h** in mice treated i.n. with anti-TNFR2 mAb (TR75.89) and TNFR2-agonist TNF_D221N/A223R_ (1 μg). Lungs were harvested 24 h later; *n* = 3 mice/group. Data are representative of two independent experiments. Graphs represent the mean with error bars indication s.e.m. *P* values determined by one-way ANOVA Tukey’s multiple comparison test **c**, **d** or unpaired student *t*-test **g**, **h**. *P < 0.05, **P < 0.001, and ***P < 0.0001.

TNFR2^−/−^ mice lack the pRelB^+^ population.^21^ Here, we found that TNFR2^fl/fl^CD11c^cre^, not the TNFR2^fl/fl^LysM^cre^ mice, lack the TNFR2^+^pRelB^+^ cDC2 (R2D2) population (Fig. 3a, b, Supplementary Fig. 3a). Thus, TNFR2 expression in DCs mediates pRelB activation. Notably, at steady state, lung cDC2 is the only TNFR2^+^ DCs population (Supplementary Fig. 3b). We hypothesized that there was an active TNFR2-pRelB signaling in the lung R2D2 population at steady state.

Indeed, blocking TNFR2 signaling with anti-TNFR2 blocking mAb inhibited pRelB expression in cDC2 (Fig. 3b) and T-regs induction in the lung (Supplementary Fig. 3c). Unexpected, blocking TNFR2 dramatically decreased the numbers of lung TNFR2^+^ pRelB^+^ cDC2 (R2D2) at steady state (Fig. 3a–c). To exclude the possibility that the anti-TNFR2 blocking mAb may interfere with the TNFR2 detection by flow cytometry, we used the TNFR2-Fc (human IgG1) fusion protein to disrupt the TNFR2 interaction with its ligand in vivo. Again, TNFR2-Fc (human IgG1) reduced R2D2 population (Fig. 3a–c). Notably, in these experiments, the numbers of lung R2D2 cells showed a more dramatic reduction (Fig. 3c) than the percentage changes (Fig. 3a). Indeed, the total numbers of lung cDC2 cells decreased in the TNFR2^fl/fl^CD11c^cre^ mice, mice treated with anti-TNFR2 blocking mAb or TNFR2-Fc likely due to the loss of the R2D2 subpopulation in lung cDC2 subset.

We further examined the Mgl2^+^/IDO-1^+^ R2D2 population and found, as expected, that blocking TNFR2 or TNFR2-Fc treatment reduced the Mgl2^+^ R2D2 number by 90% (Fig. 3d). Thus, the lung R2D2, including the T-regs-inducing Mgl2^+^ R2D2 subset, depends on the constitutive TNFR2 signaling.

TNFR2 binds specifically to transmembrane TNF (tmTNF).^32^ To identify TNF-expressing lung cells at steady state, we first did intracellular TNF staining to detect total cellular TNF, including tmTNF and soluble TNF. We found that the CD45^−^ lung cells were the main TNF^+^ lung cells at steady state (Fig. 3e). Next, we used the TNFR2-Fc (human IgG1) fusion protein to detected TNFR2 ligands, i.e., tmTNF. TNFR2 ligands were mainly on CD45^−^EP-CAM^+^ lung epithelial cells at steady state (Fig. 3f). We reasoned that, at steady state, tmTNF on lung epithelial cells engages TNFR2 on R2D2 to maintain its presence in the lung.

To mimic the effect of tmTNF that signals through TNFR2 specifically, we made a TNF_D221N/A223R_ mutant. The TNF_D221N/A223R_ mutant only binds to TNFR2,^33^ thus serves as a TNFR2-specific agonist. TNF functions as a trimer.^34^ TNF_D221N/A223R_ is a monomer. We, thus, aggregated TNFR2 in vivo with the nonblocking TNFR2 mAb followed by the addition of TNF_D221N/A223R_ to activate TNFR2 signaling. Intranasal administration of a nonblocking anti-TNFR2 mAb (TR75.89) and the TNF_D221N/A223R_ increased R2D2 population (Fig. 3g). Importantly, we found increased Ki67^+^ cells in the R2D2 population in mice treated with tmTNF (Fig. 3h), indicating the enhanced proliferation of R2D2 cells by TNFR2 engagement. As a negative control, intranasal administration of TNF_D221N/A223R_ did not enhance Ki67 expression in the cDC1 population, which do not express TNFR2 (Supplementary Fig. 3d).

In conclusion, the steady-state lung R2D2 population, including the Mgl2^+^/IDO-1^+^R2D2, is a specialized DC subset adapted to the lung microenvironment and needs the constitutive tmTNF-TNFR2 signaling for its existence.

### Constitutive IFNAR1 signaling in Mgl2^+^R2D2 cells drives lung T-regs induction

**Fig. 4:**
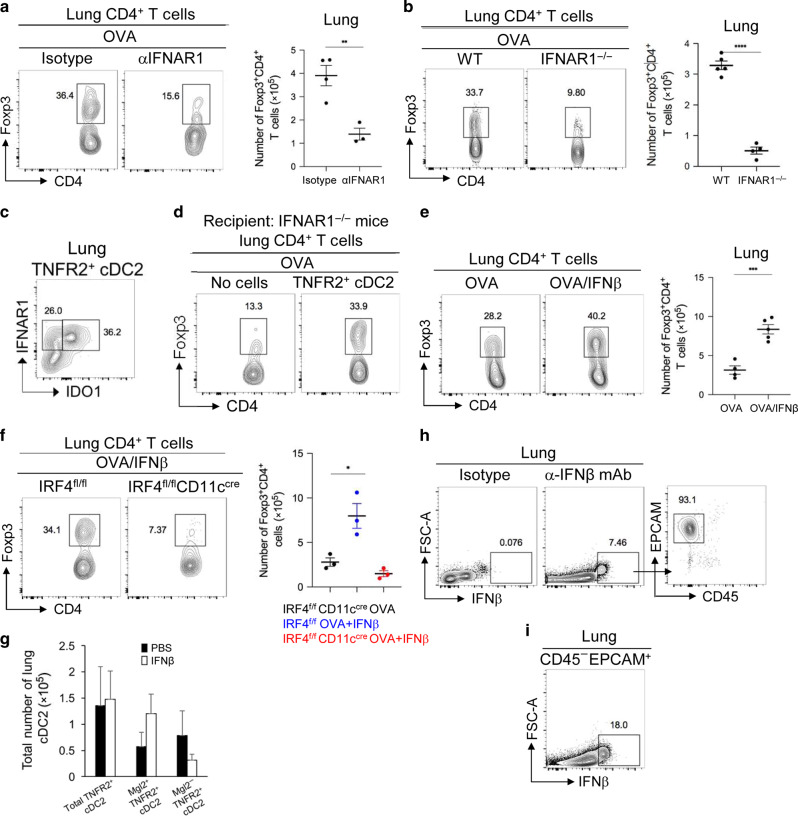
IFNβ-IFNAR1 signaling in iR2D2 cells promotes lung T-regs induction. **a** Flow cytometry analysis (left) and quantification (right) of lung TNFR2^+^ cDC2 in WT mice treated i.n. with isotype (20 µg) or anti-IFNAR1 blocking antibody (20 μg). Lungs were harvested 24 h later; *n* = 3–4 mice/group. Data are representative of three independent experiments. **b** Flow cytometry analysis (left) and quantification (right) of lung T-regs in WT treated i.n. with isotype (20 µg) or anti- IFNAR1 blocking antibody (20 μg) and one dose of OVA (1 μg) i.n. Lungs were harvested on day 14; *n* = 4–5 mice/group. Data are representative of three independent experiments. **c** Flow cytometry analysis of TNFR2^+^ cDC2 at steady state; *n* = 3 mice/group. Data are representative of two independent experiments. **d** IFNAR1^−/−^ mice were adoptively transferred (i.n.) with lung TNFR2^+^ cDC2 and one dose of OVA (1 μg) i.n. Flow cytometry analysis of T-regs on day 14; *n* = 3 mice/group. Data are representative of two independent experiment. **e** Flow cytometry analysis (left) and numbers (right) of T-regs in mice treated i.n. with OVA (1 μg) or IFNβ (200 ng). Lungs were harvested on day 14; *n* = 3–5 mice/group. Data are representative of two independent experiments. **f** Flow cytometry analysis (left) and absolute numbers (right) of T-regs in IRF4^fl/fl^ and IRF4^fl/fl^CD11c^cre^ mice treated i.n. with OVA (1 μg) or OVA (1 μg) and IFNβ (200 ng). Lungs were harvested on day 14; *n* = 3 mice/group. Data are representative of two independent experiments. **g** Numbers of Mgl2^+^TNFR2^+^ and Mgl2^−^TNFR2^+^ cDC2 in mice treated i.n. with PBS or IFNβ (200 ng); *n* = 3 mice/group. Data are representative of two independent experiment. **h**–**i** Flow cytometry analysis of IFNβ expression at steady state by the anti-IFNβ mAb (clone D2J1D); *n* = 3 mice/group. Data are representative of two independent experiments. Graphs represent the mean with error bars indication s.e.m. *P* values determined by one-way ANOVA Tukey’s multiple comparison test **f** or unpaired student *t*-test **a**, **b**, **e**. *P < 0.05, **P < 0.001, and ***P < 0.0001.

IDO-1^+^, not IDO-1^−^, R2D2 cells induce lung T-regs. However, we found that intranasal administration of an anti-IFNAR1 blocking mAb inhibited T-regs induction in the lung (Fig. 4a), but did not affect IDO-1 expression in R2D2 cells (Supplementary Fig. 4a). Similarly, IFNAR1^−/−^ mice failed to generate lung T-regs in response to OVA (Fig. 4b) but retained the R2D2 population, including the Mgl2^+^ R2D2 (Supplementary Fig. 4b) population in the lung. Thus, IDO-1 or Mgl2 expression in R2D2 is not sufficient for T-regs induction. Rather, IFNAR1 signaling is critical.

R2D2 has the highest expression of IFNAR1 among lung DCs subsets (Supplementary Fig. 4c, d) at steady state. In the R2D2 population, all IDO-1^+^ R2D2 cells express high IFNAR1 compared to the IDO-1^−^ R2D2 (Fig. 4c). Furthermore, steady-state R2D2 cells have elevated pSTAT1(Y701) signal that were further enhanced by intranasal IFNβ treatment but lost after anti-IFNAR1 treatment (Supplementary Fig. 4e).

To establish that R2D2 cell-intrinsic IFNAR1 signaling promotes lung T-regs induction, we adoptively transferred (i.n.) WT R2D2 into IFNAR1^−/−^ mice and treated the recipient mice with OVA. After 14 days, we found that IFNAR1^−/−^ mice receiving WT R2D2 cells restored T-regs induction in the lung (Fig. 4d). Thus, R2D2 cell-intrinsic IFNAR1 signaling is sufficient for lung T-regs induction.

Indeed, intranasal administration of IFNβ enhanced T-regs induction in the lung (Fig. 4e). Importantly, IFNβ did not induce T-regs in IRF4^fl/fl^CD11c^cre^ mice, suggesting IFNβ acts on cDC2 to induce T-regs (Fig. 4f). Interestingly, the intranasal administration of IFNβ did not increase the total number of lung R2D2 cells (Fig. 4g). Rather, IFNβ treatment tend to increase cell numbers of Mgl2^+^ R2D2, while decreased numbers of Mgl2^−^ R2D2 (Fig. 4g) suggesting IFNβ treatment in vivo may turn Mgl2^−^ into T-regs-inducing Mgl2^+^ R2D2. In light of the critical role of R2D2-intrinsic IFANR1 signaling in T-regs induction, we named the T-regs-inducing Mgl2^+^/IDO-1^+^ R2D2 as IFNAR1^hi^ R2D2 (iR2D2).

Last, we wanted to determine the cellular source of IFNβ in the lung that generates iR2D2 at steady state. Using intracellular IFNβ stain, we found that at steady state, the Epcam^+^CD45^−^ lung epithelial cells were the sole IFNβ-producing cells (Fig. 4h). Intriguingly, only ~18% EPCAM^+^CD45^−^ lung epithelial cells were IFNβ^+^ suggesting a selected population of lung epithelial cells are responsible for IFNβ production at steady state (Fig. 4i).

### IFNβ activates TGFβ1 in iR2D2 and drives lung T-regs induction

**Fig. 5:**
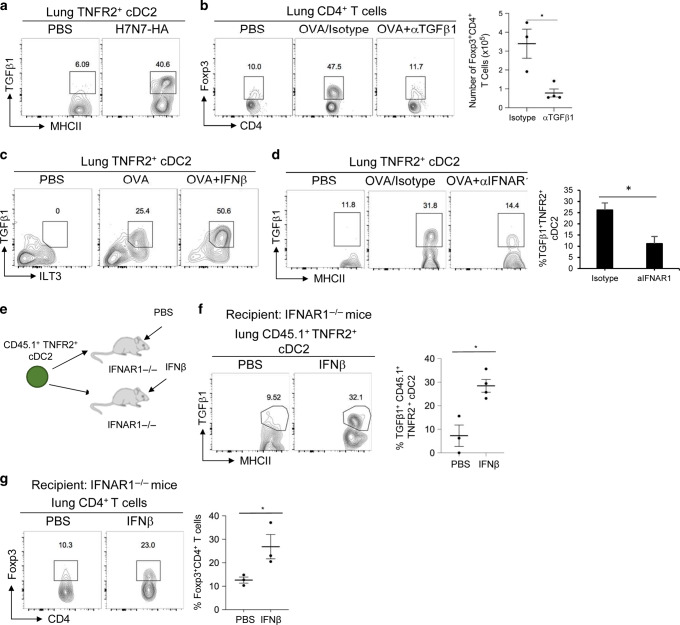
IFNβ-IFNAR1-TGFβ1 signaling axis in iR2D2 promotes lung T-regs induction. **a** Flow cytometry analysis of TNFR2^+^ cDC2 treated i.n. with PBS or H7N7-HA (1 μg). Lungs were harvested 24 h later; *n* = 3 mice/group. Data are representative of two independent experiments. **b** Flow cytometry analysis (left) and numbers (right) of T-regs in mice treated with PBS, OVA (1 μg)/isotype control, or OVA (1 μg) and anti-TGFβ1 neutralizing antibody (75 μg); *n* = 3–4 mice/group. Lungs were harvested on day 14. Data are representative of two independent experiments. **c** Flow cytometry analysis of TGFβ1 production by TNFR2^+^ cDC2 in mice treated i.n. with PBS, OVA (1 μg), or OVA (1 μg) and IFNβ (200 ng). Lungs were harvested 24 h later; *n* = 3 mice/group. Data are representative of three independent experiments. **d** Flow cytometry analysis of TGFβ1 production by TNFR2^+^ cDC2 in mice treated with PBS, OVA (1 μg)/isotype control, or OVA (1 μg) and anti-IFNAR1 blocking antibody (20 μg). Lungs were harvested 24 h later; *n* = 3 mice/group. Data are representative of two independent experiments. **e** Experimental design for adoptive transfer. **f**–**g** IFNAR1^−/−^ mice were adoptively transferred (i.n.) with lung TNFR2^+^ cDC2 and treated with PBS or IFNβ (200 ng). Flow cytometry analysis of TGFβ1 production by TNFR2^+^ cDC2 **f** and T-regs **g**; *n* = 3 mice/group. Data are representative of two independent experiments. Graphs represent the mean with error bars indication s.e.m. *P* values determined by unpaired student *t*-test **b**, **f**, **g**. *P < 0.05, **P < 0.001, and ***P < 0.0001.

How does the IFNAR1 signaling activate the tolerogenic program in the iR2D2 cells? pT-regs are generated by TGFβ1.^23–25^ R2D2 produced TGFβ1 in response to H7N7-HA (Fig. 5a). Neutralizing total TGFβ1 led to the loss of T-regs induction in the lung (Fig. 5b).

The induction of TGFβ1 in R2D2 depends on IFNβ-IFNAR1 signaling. Intranasal administration of IFNβ increased TGFβ1 in R2D2 (Fig. 5c), while anti-IFNAR1 blocking mAb inhibited OVA-induced TGFβ1 production by R2D2 (Fig. 5d). Notably, anti-IFNAR1 mAb did not affect OVA-induced TGFβ1 production in cDC1, moDCs, or AMs (Supplementary Fig. 5a). Block IFNAR1 inhibited OVA-induced T-regs induction (Fig. 4a). Thus, TGFβ1 production by cDC1, AM, or moDCs were not sufficient for the induction of T-regs in the lung.

To establish that IFNβ acted on R2D2 to induce TGFβ1 expression, we adoptively transferred (i.n.) the lung R2D2 cells from the CD45.1^+^ mice into IFNAR1^−/−^ mice and treated (i.n.) the recipient IFNAR1^−/−^ mice with IFNβ (Fig. 5e). We found that IFNβ administration induced TGFβ1 in the transferred CD45.1^+^ cells (Fig. 5f). Furthermore, IFNβ restored T-regs induction in the IFNAR1^−/−^ mice received CD45.1^+^ R2D2 cells (Fig. 5g), suggesting that IFNβ acted on lung R2D2 cells to generate TGFβ1 and lung T-regs induction. Taken together, IFNAR1 signaling in iR2D2 produces TGFβ1 and induces T-regs in the lung.

### R2D2 cells promote T_H_2 responses in HDM-induced asthmatic mice

cDC2 mediates HDM-induced asthma.^13,16,18^ Recent studies indicated that a Klf4^+^/Mgl2^+ 13^, RelB^+^ cDC2 subset^10^ mediates HDM-induced T_H_2 responses. iR2D2 expresses Mgl2, RelB. We suspected that the lung R2D2 population may be plastic and promote T_H_2 response in HDM-induced asthma.

**Fig. 6:**
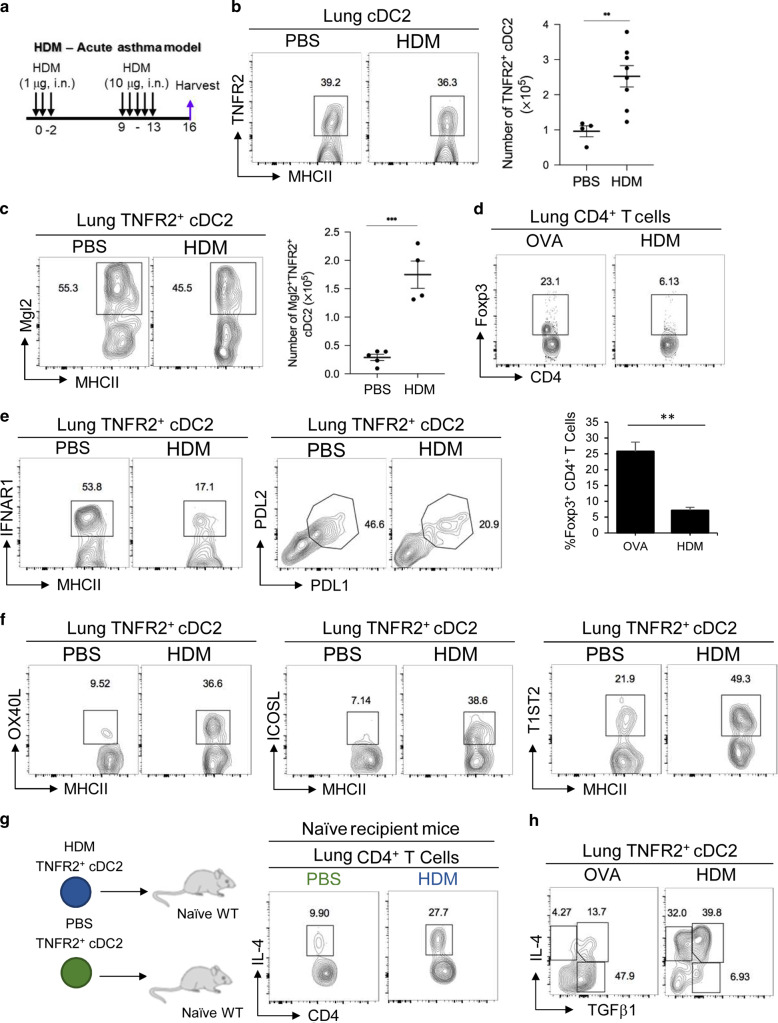
R2D2 promotes T_H_2 responses in HDM mice. **a** Experimental protocol for HDM-induced acute asthma. **b** Flow cytometry analysis (left) and numbers (right) of TNFR2^+^ cDC2 in PBS or HDM-induced asthmatic WT mice; *n* = 2–4 mice/group. Data were compiled from two independent experiments. **c** Flow cytometry analysis of Mgl2^+^TNFR2^+^ cDC2 in PBS or HDM-induced asthmatic WT mice; *n* = 4–5 mice/group. Data are representative of three independent experiments. **d** Flow cytometry analysis of T-regs in OVA (1 µg) treated (left) and HDM-induced asthmatic WT mice (right); *n* = 3 mice/group. Data are representative of three independent experiments. **e**–**f** Flow cytometry analysis of TNFR2^+^ cDC2 in HDM-induced asthmatic WT mice; *n* = 3 mice/group. Data are representative of three independent experiments. **g** Experimental design for adoptive transfer (top). Flow cytometry analysis of IL-4 production by lung CD4^+^ T cells; *n* = 3mice/group. Data are representative of two independent experiments. **h** Flow cytometry analysis of R2D2 in OVA (1 µg) treated (left) and HDM-induced asthmatic WT mice (right); *n* = 3 mice/group. Data are representative of three independent experiments. Graphs represent the mean with error bars indication s.e.m. *P* values determined by unpaired student *t*-test **b**. *P < 0.05, **P < 0.001, and ***P < 0.0001.

First, we generated the HDM-induced asthmatic mice (Fig. 6a). HDM-treated mice generated HDM-specific IgE and IgG1 (Supplementary Fig. 6a), lung inflammation determined by hematoxylin and eosin (H&E) stain (Supplementary Fig. 6b), eosinophils infiltration in the lung (Supplementary Fig. 6c), and T_H_2 dominant cytokines in draining LNs (Supplementary Fig. 6d). Next, we examined the iR2D2 population in the asthmatic mice. We found increased numbers of total R2D2 cells in the asthmatic cells (Fig. 6b). The Mgl2^+^/IDO-1^+^ R2D2 population increased as well in the HDM-induced asthmatic mice (Fig. 6c, Supplementary Fig. 6e). However, no T-regs were generated in HDM mice (Fig. 6d). The R2D2 cells from HDM-induced mice also downregulated tolerogenic markers PD-L1, PD-L2, and, critically, IFNAR1 expression (Fig. 6e). In contrast, R2D2 cells from the HDM mice increased immunogenic markers OX40L, ICOSL, and T1/ST2 (Fig. 6f).

Last, adoptive transfer (i.n.) R2D2 from HDM mice into naive mice generated T_H_2 response in vivo (Fig. 6g), which was different from the T-regs-inducing R2D2 at steady state (Fig. 2d). Comparing the tolerogenic R2D2 (iR2D2) from OVA-treated mice with the T_H_2-promoting R2D2 from the HDM mice, the tolerogenic R2D2 expresses TGFβ1 while the immunogenic R2D2 expresses IL-4 and IL-4/TGFβ1 (Fig. 6h). We concluded that the lung R2D2 population is plastic and can promote T-regs or T_H_2 responses depending on the environmental cue, e.g., IFNβ or HDM.

### Human lungs have a phenotypically similar plastic iR2D2 population

**Fig. 7:**
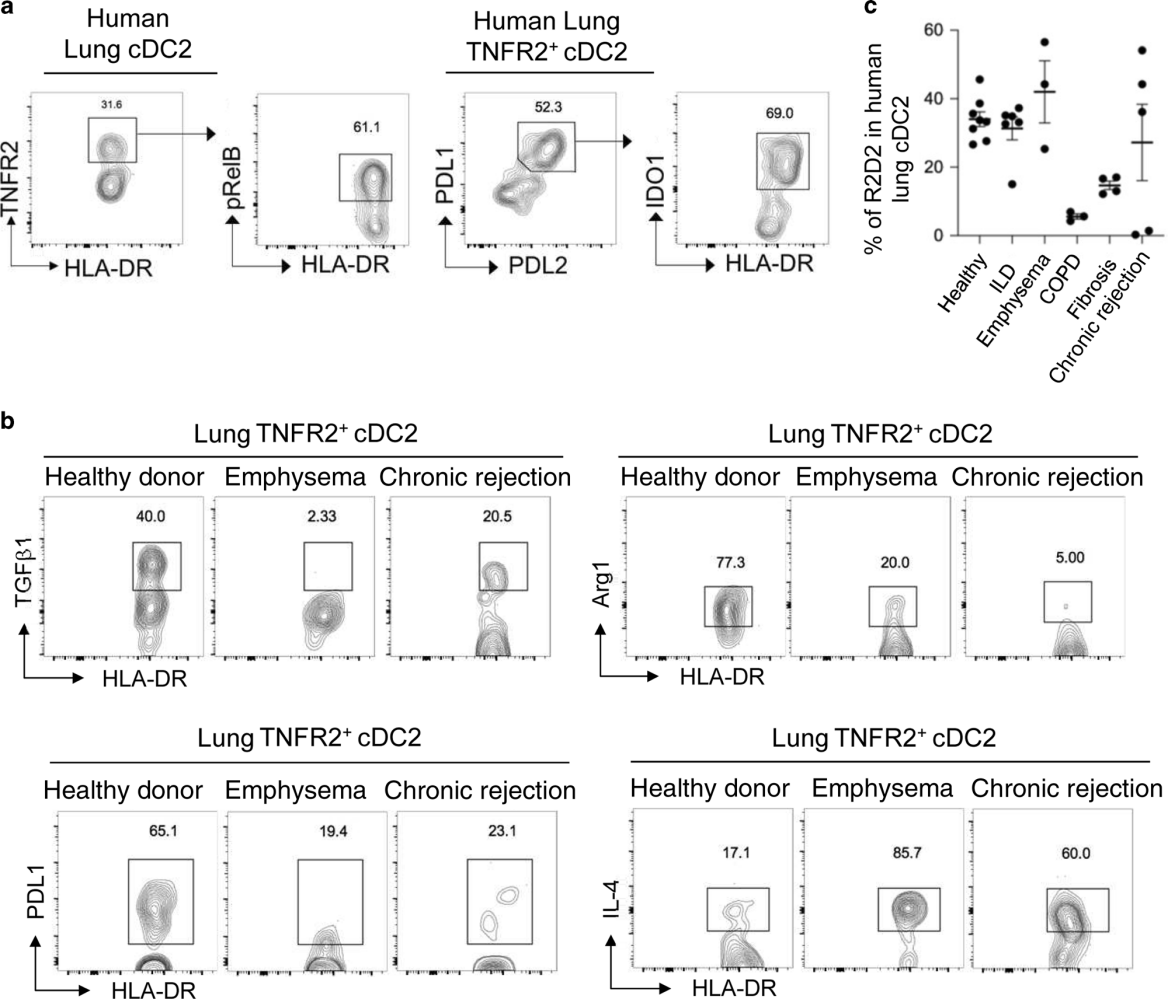
Lung TNFR2^+^ cDC2 in healthy and lung diseases patients. **a** Flow cytometry analysis of pulmonary cDC2 subpopulation in healthy human lungs. Data are representative of eight independent experiments. **b** Flow cytometry analysis of TNFR2^+^cDC2 in donor, emphysema, and chronic-rejected human lungs. Data are representative of three independent experiments. **c** Frequency of TNFR2^+^ cDC2 in diseased human lungs. Data were compiled from multiple independent experiments. Graphs represent the mean with error bars indication s.e.m.

We reasoned that healthy human lung should have a functionally similar tolerogenic DC subset. Little is known about human lung cDC2 subsets in healthy individuals or patients. We sampled healthy donor lungs that were transplanted into lung patients and identified the same TNFR2^+^pRelB^+^IDO-1^+^PD-L1^+^PD-L2^+^ cDC2 population in the healthy human lungs (Fig. 7a, Supplementary Fig. 7a, b). The healthy human lung iR2D2 cells constitutively express TGFβ1 and Arg-1 (Fig. 7b) as well.

We also identified IDO-1^+^ R2D2 population in lung explants from emphysema, interstitial lung disease patients (Fig. 7c). However, R2D2 cells from lung patients had decreased TGFβ1, Arg-1, and PD-L1, but increased IL-4 expression (Fig. 7b) similar to the R2D2 from the asthmatic mice. Notably, lung explants from chronic obstructive pulmonary disease patients had very little R2D2 cells (Fig. 7c). We conclude that the phenotypically similar R2D2 cells can be found in healthy human lungs and some lung disease patients.

## Discussion

Here, we identified a lung iR2D2 as the tolerogenic DC generating lung T-regs and preventing lung inflammation at steady state. Our conclusion is based exclusively on studies in the lung in vivo. Our conclusion is different from previous reports, suggesting pDCs,^2^ cDC1^14^, or macrophage^15^ induces T-regs in the lung. We showed that mice lacking the iR2D2 population by gene ablation or antibody depletion failed to generate lung T-regs, while intranasal administration of IFNβ induced R2D2-dependent lung T-regs. Notably, the data present here could not exclude a role for lung macrophage in inducing lung T-regs. Thought it is unlikely macrophage direct prime T cells, lung macrophage could cooperate with R2D2 to generate T-regs in the lung.^15^

The lung iR2D2 population is plastic. iR2D2 is a subpopulation of cDC2. Previous studies established that cDC2 mediates T_H_2,^10,13,16^ T_H_17^19,20^ responses. Meanwhile, Mellman’s group showed that IRF4^fl/fl^CD11c^cre^ mice had impaired peripheral tolerance.^35^ Most recently, Berlin’s group identified a PD-L2^+^CD11b^+^ dermal DCs that can elicit T_H_2 responses or prime T-regs.^36^ We also showed that R2D2 promotes T_H_ responses of the mucosal adjuvant cyclic di-GMP in vivo*.*^21^ We propose that DCs at the barrier surface, influenced by their microenvironment, are plastic-inducing T-regs, maintaining peripheral tolerance at steady state but promoting immunogenic responses during pathogenic conditions. Indeed, Agace’s group showed that CD103^+^ DCs in the gut has a dual role in tolerogenic and immunogenic T-cell responses.^4^

The tolerogenic iR2D2 population is a product of two constitutive signals from its microenvironment: IFNβ-IFNAR1 and tmTNF-TNFR2. IFNβ promote T-regs in the gut.^37^ Using a TNFR2 agonist, we showed that TNFR2 signaling promoted R2D2 proliferation. TNFR2 expression defines a highly immunosuppressive T-regs found in tumor microenvironment promoting cancer cell survival and tumor growth.^38^ Similar to our R2D2 cells, TNFR2 activation stimulates the proliferation of TNFR2^+^ T-regs.^39^ Antagonistic antibodies against human TNFR2 inhibited TNFR2^+^ T-regs proliferation.^39^ The underlying molecular mechanism of TNFR2 signaling induced cell proliferation is unknown.

R2D2 cells with active IFNAR1 signaling become iR2D2. iR2D2 intrinsic IFNβ signaling is necessary and sufficient for lung T-regs induction and the production of TGFβ1. However, the molecular pathway by which IFNβ-IFNAR1 signaling leading to TGFβ1 production is unknown. IFNβ has an immunomodulatory effect. In the clinic, IFNβ (Avonex®, Rebif®) has been used to treat relapsed multiple sclerosis for over 20 years. Nevertheless, the in vivo mechanism and targeted cells of IFNβ treatment remain poorly defined.^40^ We propose that lung R2D2 or R2D2-like cells in other peripheral organs^41^ are the effector cells for the immunomodulatory function of IFNβ in vivo. Noteworthy, IFNα is not approved for multiple sclerosis. Future studies should also determine if IFNβ activates a unique IFNAR1 signaling^42^ in iR2D2 cells to control the tolerogenic program at steady state.

**Fig. 8:**
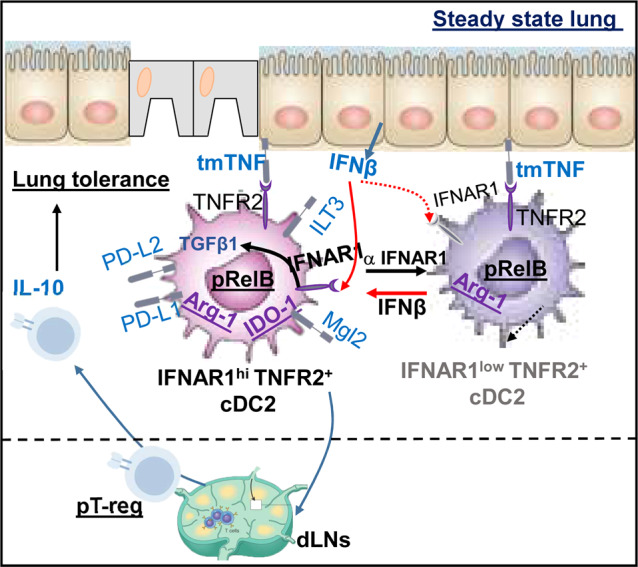
Model. Lung epithelium IFNβ/iR2D2/T-regs axis controls lung tolerance at steady state.

Lung epithelial cells are a major source of tmTNF and the sole producer of lung IFNβ at the steady state. Impaired bronchial epithelial cells (BECs) IFNβ production has been well documented in asthmatic patients.^43^ We thus propose a new paradigm that at steady state, lung epithelial cells express tmTNF to keep R2D2 alive and continuously secret IFNβ to condition R2D2 to generate lung T-regs and prevent lung inflammation (Fig. 8). Different from the current lung-epithelial cells-DCs-T_H_2 axis during inflammation,^44^ the lung epithelial cells IFNβ-iR2D2-T-regs axis here is tolerogenic and needed to be constitutively active. Loss of tmTNF or IFNβ will result in the loss of iR2D2 and lung mucosal tolerance. Indeed, BECs from asthma patients are biased toward higher Thymic stromal lymphopoietin (TSLP) and lower IFNβ production.^45^ Commensal microbiota drive tonic IFNβ production by lung epithelial cells that protect host from respiratory viral infection.^46,47^ It is worth investigating if commensal microbiota can also protect host from asthma by driving lung IFNβ and enhancing steady-state lung mucosal tolerance.

The plasticity of R2D2 cells makes them an ideal immunotherapy target for chronic inflammatory diseases. We showed that IFNβ administration could enhance lung T-regs induction. A phase II clinical trial of inhaled IFNβ for virus-induced asthma exacerbation showed promising results (clinicaltrials.gov, identifier NCT 01126177).^48,49^ Translationally, targeting IFNβ directly to R2D2 will limit the toxicity of IFNβ and improve its efficacy. On the other hand, for studies exploring the therapeutic potential of ex vivo generated tolerogenic DCs, caution should be taken. Due to the plasticity of tolerogenic DCs, these ex vivo generated tolerogenic DCs maybe plastic and could be reconditioned by the inflammatory milieu and become immunogenic in vivo.

In summary, we identified iR2D2 as the tolerogenic lung DC population and surprisingly found it is plastic. This newly discovered DC plasticity can lead to novel anti-inflammatory therapy for lung diseases.

## Methods

### Key resources table

**Table:** 

Reagent or resource	Source	Identifier
Antibodies		
Anti-mouse CD4-PE/Cy7 (clone: GK1.5)	BioLegend	Cat. #100422
Anti-mouse IFNγ-PerCP/Cy5.5 (clone: XMG1.2)	BioLegend	Cat. #505822
Anti-mouse IL-4-APC (clone: 11B11)	BioLegend	Cat. #504106
Anti-mouse IL-17a-PE (clone: TC11-1810.1)	BioLegend	Cat. #506903
Anti-mouse CD44-Alexa Fluor 700 (clone: IM7)	BioLegend	Cat. #103025
Anti-mouse CD45-PercP/Cy5.5 (clone: 30-F11)	Biolegend	Cat. #103131
Anti-mouse Foxp3-Pacific Blue (clone: MF-14)	BioLegend	Cat. #26410
Anti-mouse MHCII(I-A/I-E)-Brilliant Violet 421 (clone: M5/114.15.2)	BioLegend	Cat. #107636
Anti-mouse MHCII(I-A/I-E)-Alexa Fluor (clone: M5/114.15.2)	BioLegend	Cat. #107622
Anti-mouse CD11c-APC/Cy7 (clone: N418)	Biolegend	Cat. #117323
Anti-mouse/human CD11b-PE/Cy7 (clone: M1/70)	BioLegend	Cat. #101216
Anti-mouse/human CD11b-Brilliant Violet 605 (clone: M1/70)	BioLegend	Cat. #101237
Anti-mouse CD64-PerCP/Cy5.5 (clone: X54-5/7.1)	BioLegend	Cat. #139307
Anti-mouse TNFR2-PE (clone:TR75-89)	BioLegend	Cat. #113405
Anti-mouse TNFR2-APC (clone: REA228)	Miltenyi Biotec	Cat. #130-104-698
Anti-mousPDL1-Brilliant Violet 421 (clone: 10F.9G2)	BioLegend	Cat. #124315
Anti-mouse PDL2-APC (clone: TY25)	BioLegend	Cat. #107210
Anti-mouse/human Arg1-FITC	RD systems	Cat. #IC5868F
Anti-mouse ILT3-PE (clone: H1.1)	BioLegend	Cat. #144904
Anti-mouse IDO1-Alexa Fluor (clone: 2E2/IDO1)	BioLegend	Cat. #654003
Anti-mouse/human pRelB-PE (clone: D41B9)	Cell Signaling Technology	Cat. #:13567
Anti-mouse/human pSTAT1 (clone: 58D6)	Cell Signaling Technology	Cat. #:9167
Anti-mouse EPCAM-PerCP/Cy5.5 (clone: G8.8)	BioLegend	Cat. #118219
Anti-mouse/human Ki67-PE (clone: 11F6)	BioLegend	Cat. #151209
Anti-mouse IFNAR1-APC (clone: MAR1-5A3)	BioLegend	Cat. #127313
Anti-mouse IFNβ-Primary (clone:D2J1D)	Cell Signaling Technology	Cat. #:974505
Anti-mouse TNF-Primary (clone:D2D4)	Cell Signaling Technology	Cat. #:11948
Anti-mouse LAP (TGFβ1)-Brilliant Violet 421 (clone:TW7-16B4)	BioLegend	Cat. #141407
Anti-mouse LAP (TGFβ1)-FITC (clone:TW7-16B4)	BioLegend	Cat. #141413
Anti-mouse CD45.1-APC (clone: A20)	BioLegend	Cat. #110713
Anti-mouse OX40L-PE (clone: RM134L)	BioLegend	Cat. #108805
Anti-mouse ICOSL-PE (clone: HK5.3)	BioLegend	Cat. #107405
Anti-mouse T1ST2-APC (clone: D1H4)	BioLegend	Cat. #146605
Anti-mouse IRF4-APC (clone: IRF4.3E4)	BioLegend	Cat. #646407
Anti-human TNFR2-APC (clone: 3G7A02)	BioLegend	Cat. #358405
Anti-human HLA-DR-APC/Cy7 (clone: L243)	BioLegend	Cat. #307617
Anti-human PDL1-Brilliant Violet 421 (clone:29E.2A3)	BioLegend	Cat. #329713
Anti-human PDL2-PE (clone: 24F.10C12)	BioLegend	Cat. #329605
Anti-human IDO1-Primary	R&D Systems	Cat. #MAB6030
Anti-human TGFβ1-PE (clone: Tw4-2F8)	BioLegend	Cat. #349603
Anti-human Arginase 1-PE (clone: 14D2C43)	BioLegend	Cat. #369703
Anti-human IL-4-PE (clone: G077F6)	BioLegend	Cat. #355003
Anti-mouse F4/80-PerCP/Cy5.5 (clone: BM8)	BioLegend	Cat. #123127
I-A(b) chicken ova 325-335 QAVHAAHAEIN APC-labeled tetramer	NIH Tetramer Core Facility	
I-A(b) human CLIP 87-101 PVSKMRMATPLLMQA (control) APC-labeled tetramer	NIH Tetramer Core Facility	
Anti-mouse Neuropilin-APC (clone: 3E12)	BioLegend	Cat. #145205
Anti-human CD1c-PE/Cy7 (clone: L161)	BioLegend	Cat. #331515
Anti-human CD14-PerCP/Cy5.5 (clone: 63D3)	BioLegend	Cat. #367109
Anti-human CD206-FITC (clone: 15–2)	BioLegend	Cat. #321103
Anti-CCR7 monoclonal antibody	R&D Systems	Cat. #MAB34477
Anti-TNFR2 monoclonal antibody (clone: TR75–54.7)	BioXcell	Cat. #BE0247
Anti-TNFR2 monoclonal antibody (clone: TR75–32.4)	Biolegend	Cat. #113202
Isotype control (clone HTK888)	Biolegend	Cat. #400931
Anti-TGFβ1 neutralizing antibody (19D8)	Biolegend	Cat. #521707
Anti-IFNAR1 monoclonal antibody (clone: MAR1-5A3)	Biolegend	Cat. #127303
Anti-rabbit IgG (H+L), F(ab′)_2_ fragment-PE	Cell Signaling Technology	Cat. #:79408
Anti-mouse IgG1-HRP	Southern Biotech	Cat. #1030-05
Anti-mouse IgE-HRP	Southern Biotech	Cat. #1110-05
Chemicals, peptides, and recombinant proteins		
Cell Activation Cocktail with Brefreldin A	Biolegend	423303
OVA	Invivogen	Cat. #vac-pova
TNFR2-Fc (human IgG1) fusion protein	SinoBiological	Cat. #50128-M02H
TNFR2-agonist (TNF_D221N/A223R_)	Creative® Biolabs	Custom made
Recombinant murine IFNβ	R&D	Cat. #8234-MB/CF
House dust mites *Dermatophagoides pteronyssinus* (HDM-Der p1)	Greer Laboratories	Cat. #XPB82D3A2.5
* Dermatophagoides farinae* (HDM-Der f1)	Greer Laboratories	Cat. #XPB81D3A2.5
PspA	BEI Resources, NIAID, NIH	NR-33178
H7N7-HA	BEI Resources, NIAID, NIH	NR-2633
H1N1-NP	SinoBiological	Cat. #11675-V08B
Foxp3/Transcription Factor Staining Buffer Set	EBioscience	Cat. #00-5523-00
Experimental models: organisms/strains		
Mouse: IRF4^fl/fl^	Jackson Laboratory	Cat. #009380
Mouse: CD11c^Cre^	Jackson Laboratory	Cat. #008068
Mouse: Batf3^−/−^	Jackson Laboratory	Cat. #013596
Mouse: CCR2^−/−^	Jackson Laboratory	Cat. #004999
Mouse: RelB^fl/fl^	Jackson Laboratory	Cat. #028719
Mouse: TNFR2^fl/fl^ [C57BL/6-Tnfrsf1b<tm1c(EUCOMM)Wtsi>/Ics]	EMMA—European Mouse Mutant Archive	Cat. #05925
Mouse: IL-10^eGFP^	Jackson Laboratory	Cat. #014530
Mouse: TNFR1^−/−^	Jackson Laboratory	Cat. #003243
Mouse: IFNAR1^−/−^	Jackson Laboratory	Cat. #028288
Mouse: CD45.1	Jackson Laboratory	Cat. #002014
Mouse: Lysm^cre^	Jackson Laboratory	Cat. #004781
Software and algorithms		
FlowJo version 10.1r1	FlowJo	http://www.flowjo.com
Prism6	GraphPad	http://www.graphpad.com

#### Mice

Age- and gender-matched mice (8–18-weeks old) were used for all experiments. C57BL/6, B6.CD45.1, *Batf3*^−/−^, *CCR2*^−/−^, *IL-10*^*GFP*^, *IRF4*^*fl/fl*^, *RelB*^*fl/fl*^, *IFNAR1*^−/−^, *CD11c*^*cre*^, *LysM*^*cre*^, and *TNFR1*^−/−^ mice on C57BL/6 background were purchased from The Jackson Laboratory. *TNFR2*^*fl/fl*^ mice were from the Euorpean Conditional Mouse Mutagenesis Program. Mice were housed and bred under pathogen-free conditions in the Animal Research Facility at the University of Florida. All mouse experiments were performed by the regulations and approval of the Institutional Animal Care and Use Committee at the University of Florida, IACUC number 201909362.

#### Reagents

Anti-TNFR2 monoclonal antibody (20 µg, TR75-54.7, BioXcell or TR75-32.4, BioLegend), anti-IFNAR1 monoclonal antibody (20 µg, MAR1-5A3, BioLegend), isotype control (20 µg HTK888, BioLegend), anti-TGFβ1 neutralizing antibody (75 µg, 19D8, BioLegend), anti-IFNβ monoclonal antibody (D2J1D, Cell Signaling, cat. no. 97450), TNFR2-Fc (human IgG1) fusion protein (2 µg, SinoBiological, cat. no. 50128-M02H), or TNF_D221N/A223R_ (1 µg, custom made by Creative® Biolabs) were administered i.n. in 40 µl phosphate-buffered saline (PBS). Recombinant mouse IFNβ (200 ng, ~240,000 IU; R&D, cat. no. 8234-MB/CF) was administered i.n. in 40 µl PBS.

The following reagent was obtained through BEI Resources, NIAID, NIH: *Streptococcus pneumoniae* family 2, clade 3 pneumococcal surface protein A (PspA UAB099) with C-terminal histidine tag, recombinant from *Escherichia coli*, NR-33179. H7 hemagglutinin (HA) protein from influenza virus, A/Netherlands/219/2003 (H7N7), recombinant from baculovirus, NR-2633. H1N1-NP was from SinoBiological (cat. no. 11675-V08B). Endotoxin-free OVA was from Invivogen (cat. no. vac-pova).

#### HDM-induced asthma

HDMs *Dermatophagoides pteronyssinus* (HDM-Der p1, Greer Laboratories, cat. no. XPB82D3A2.5) or *Dermatophagoides farinae* (HDM-Der f1, Greer Laboratories, cat. no. XPB81D3A2.5) was suspended in endotoxin-free PBS at a concentration of 5 mg/ml. HDM was freshly prepared by mixing equal parts of HDM-Der p1 and HDM-Der f1 in PBS. To induce asthma, mice were sensitized i.n. with three daily doses of 1 µg HDM on days 0–2 and were later challenged with 10 µg of HDM i.n. on days 9–13. BAL fluid, blood, lungs, and medLNs were collected on day 16. BAL fluid was collected in 0.7–1 ml PBS and blood was collected through cardiac puncture.

#### Lung histology

Lungs were fixed in 10% formalin, paraffin embedded, and cut into 4-µm sections. Lung sections were then stained for H&E. All staining procedures were performed by the histology core at the University of Florida.

#### Isolation of lung cells

Cells were isolated from the lung as previously described.^21^ The lungs were perfused with ice cold PBS and removed. Lungs were digested in DMEM containing 200 μg/ml DNase I (Roche, 10104159001), and 25 μg/ml Liberase TM (Roche, 05401119001) at 37 °C for 2 h. Red blood cells were then lysed and a single cell suspension was prepared by filtering through a 70-µm cell strainer.

#### HDM ELISA

HDM-specific IgG1 and IgE were measured by enzyme-linked immunosorbent assay (ELISA) in the serum of HDM-treated mice. Secondary Abs used were anti-mouse IgG1-HRP (Southern Biotech, cat. no.1070–05) and anti-mouse IgE-HRP (Southern Biotech, cat. no.1110–05). To measure HDM-specific T-cell responses, lung cells were restimulated with 25 µg/ml of HDM for 4 days. IL-5 cytokines were measured in the supernatant by ELISA.

#### Flow cytometry

Single cell suspensions were stained with flourescent-dye-conjugated antibodies in PBS containing 2% fetal bovine serum and 1 mM EDTA. Surface stains were performed at 4 °C for 20 min. For intracellular cytokine or transcription factor stainings of murine and human cells, cells were fixed and permeabilized with the Foxp3 staining buffer set (eBioscience, cat. no 00-5523-00). CD4^+^FoxP3^+^ T-regs in the lung were analyzed 2 weeks after the treatment. Data were acquired on a BD LSRFortessa and analyzed using FlowJo software package (FlowJo, LLC). Cell sorting was performed on the BD FACSAriaIII Flow Cytometer and Cell Sorter.

#### Adoptive transfer

cDC2 subpopulations were sorted from the lungs of naive or HDM-treated mice with a FACSAriaIII flow cytometer. cDC2 were identified as MHCII^+^CD11c^+^ CD11b^+^ CD64^−^. cDC2 subsets were defined as MHCII^+^CD11c^+^CD11b^+^TNFR2^+^ and MHCII^+^CD11c^+^ CD11b^+^ TNFR2^−^.^21^ A total of 500,000 cells were administered i.n. into recipient mice. DCs in the lungs were analyzed 24 h later. T cells in the lung were analyzed 14 days later.

### Human lung explants

Human lung explants were procured at the Lung Transplant Center, Division of Pulmonary, Critical Care and Sleep Medicine, Department of Medicine, University of Florida. Donor and patients consent for a research protocol (UF Lung Transplant Tissue/Databank (IRB201501133)). Healthy donor lungs were surgically removed postmortem, perfused, small pieces were cut from the right middle and lower lobes for research purpose, and stored in cold Perfadex® at 4 °C for no more than 12 h before processing. Ex planted lungs from emphysema lung transplant patients were stored in cold Perfadex® at 4 °C for no more than 12 h before the process. No lung explants were procured from prisoners.

### Statistical analysis

All data are expressed as means ± s.e.m. Statistical significance was evaluated using Prism 6.0 software. One-way analysis of variance (ANOVA) was performed with post hoc Tukey’s multiple comparison test, Mann–Whitney *U*-test, or Student’s *t*-test applied as appropriate for comparisons between groups. A *P* < 0.05 was considered significant.

## Supplementary information

Supplementary Figures
