# Supplementary material for: Lung IFNAR1hi TNFR2+ cDC2 promotes lung regulatory T cells induction and maintains lung mucosal tolerance at steady state
Source: Mucosal Immunol. 2020 Jan 20;13(4):595–608. doi: 10.1038/s41385-020-0254-1 (PMC7311323; doi:10.1038/s41385-020-0254-1)
Supplement: Supplementary file 1 — Supplementary Figures [file 41385_2020_254_MOESM1_ESM.pdf]

Supplemental Figures:

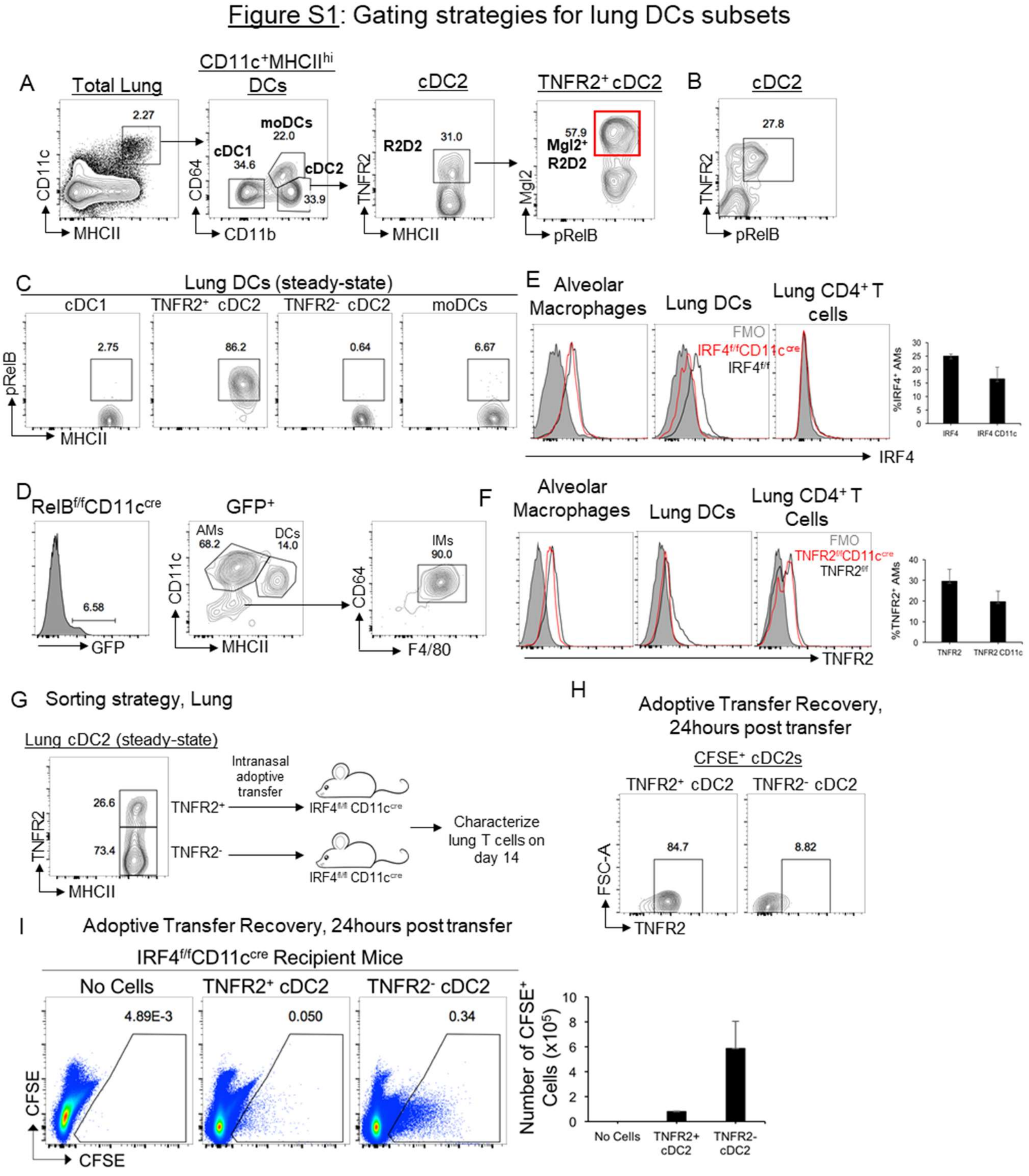

Supplemental Figure 1. Gating strategies for lung DCs subsets. A-B. Gating strategy for lung

DCs. cDC1 are $MHCII^{hi}CD11c^{+}CD11b^{-}CD64^{-}$, moDCs are $MHCII^{hi}CD11c^{+}CD11b^{+}CD64^{+}$, and cDC2 are $MHCII^{hi}CD11c^{+}CD11b^{+}CD64^{-}$. R2D2 are $MHCII^{hi}CD11c^{+}CD11b^{+}CD64^{-}TNFR2^{+}$ and further characterized based by Mgl2 expression. **C.** Flow cytometry analysis of pRelB expression in lung DCs. **D.** Flow cytometry analysis of GFP-expression in $RelB^{f/f}CD11c^{cre}$ mice at steady-state. **E.** Histograph of IRF4 expression in $IRF4^{f/f}$ and $IRF4^{f/f}CD11c^{cre}$ mice at steady-state. . n=4 mice/group. Data are representative of three independent experiment. **F.** Histograph of TNFR2 expression in $TNFR2^{f/f}$ and $TNFR2^{f/f}CD11c^{cre}$ mice at steady-state. . n=3mice/group. Data are representative of three independent experiment. **G**. Experimental scheme for the adoptive transfer of $TNFR2^{+}$ and $TNFR2^{-}$ cDC2 into $IRF4^{f/f}CD11c^{cre}$ mice. **H-I**. A total of 500,000 CFSE-labelled $TNFR2^{+}$ or $TNFR2^{-}$ cDC2s were transferred into recipient mice. Phenotypic analysis of CFSE-labelled lung cDC2 transferred into $IRF4^{f/f}CD11c^{cre}$ mice 24 hours post transfer. n=3mice/group. Data are representative of three independent experiment. Graphs represent the mean with error bars indication s.e.m. *P* values determined by unpaired student *t*-test

Figure S2: Innocuous inhaled protein antigens induce peripheral T-regs in the lung

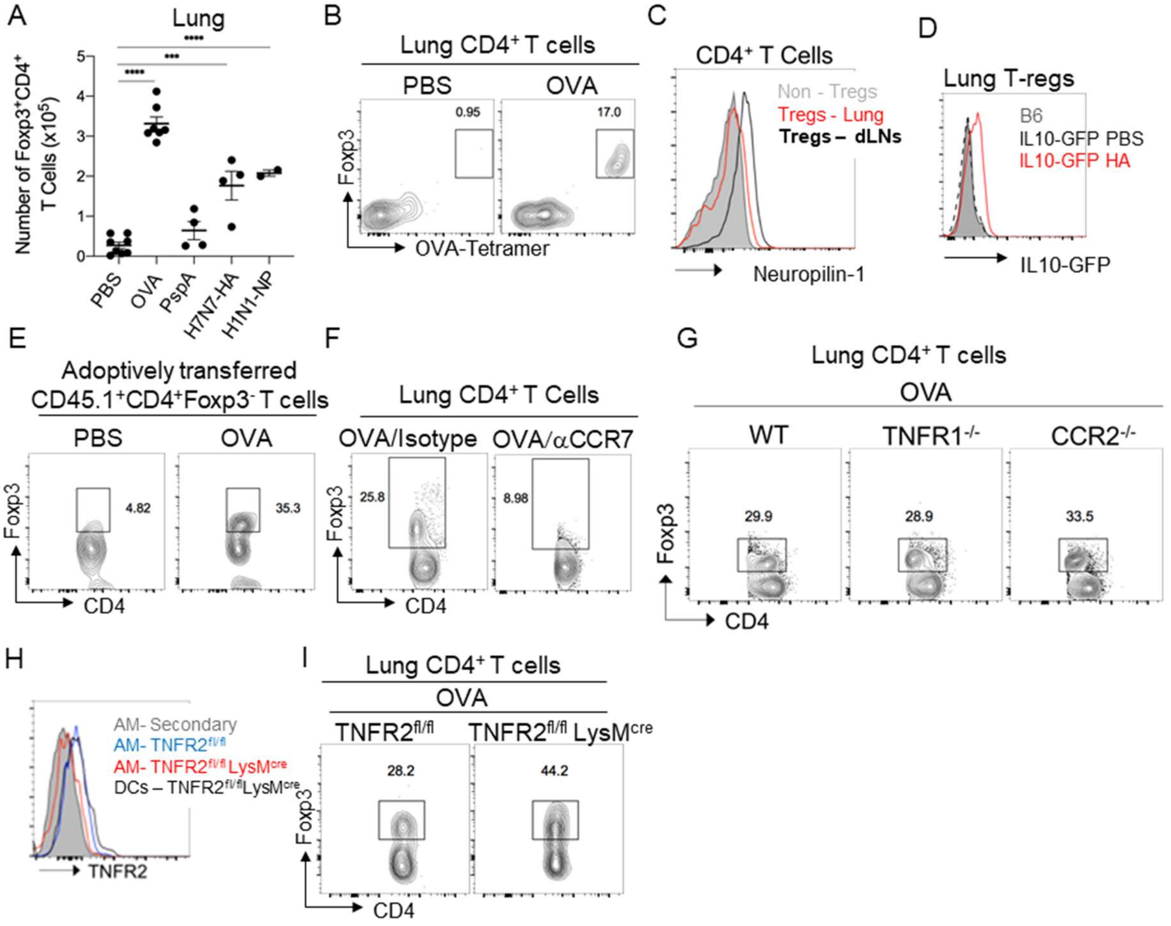

Supplemental Figure 2. Innocuous inhaled protein antigens induce peripheral T-regs in the lung. A. Numbers of $CD4^+Foxp3^+$ T-regs cells in WT mice treated with one dose (1µg) of OVA, PspA, H7N7-HA, or H1N1-NP. Lungs were harvested on day 14. Data were compiled from two independent experiments. **B.** Flow cytometry analysis of antigen-specific $CD4^+$ T-regs in the lungs of WT mice treated with PBS or OVA (1µg) *i.n.* Cells were first gated on $CD4^+$ T cells. n=3mice/group. Data were representative of two independent experiments. **C.** Flow cytometry analysis of neuropilin-1 expression on lung and dLNs T-regs. n=3 mice/group. Data were representative of two independent experiments. **D.** Representative histograph showing IL-10-GFP expression in mice treated with PBS or HA (1µg). n=3 mice/group. Data were representative of

two independent experiments. **E.** Naïve $CD4^+Foxp3^-$ T cells were sorted from the spleen of $CD45.1^+$ mice. Cells were transferred into WT intravenously (*i.v.*). Recipient mice were treated with PBS or OVA (1µg) *i.n.* Lungs were harvested on day 14. n=3 mice/group. Data were representative of two independent experiments. **F.** Flow cytometry analysis of T-regs in mice treated with OVA (1µg) *i.n.* and isotype control or anti-CCR7 (20µg). n=3 mice/group. Data were representative of two independent experiments. **G.** Flow cytometry plots of T-regs in WT, $TNFR1^{-/-}$ and $CCR2^{-/-}$ mice treated with one dose of OVA (1µg) (*i.n.*). Lungs were harvested on day 14. n=3 mice/group. Data were representative of two independent experiments. **H.** Flow cytometry analysis of TNFR2 expression in alveolar macrophage (AM) and DCs from indicated mice. n=3mice/group. Data are representative of two independent experiments. **I.** Flow cytometry analysis of T-regs in $TNFR2^{f/f}$ and $TNFR2^{f/f}LysM^{cre}$ mice treated with OVA (1µg) *i.n.* n=3 mice/group. Data were representative of two independent experiments. Graphs represent the mean with error bars indication s.e.m. P values determined by ANOVA (A). *P<0.05, **P<0.001, ***P<0.0001.

Figure S3: Tonic TNFR2 signaling is required for the presence of the R2D2 population in the lung

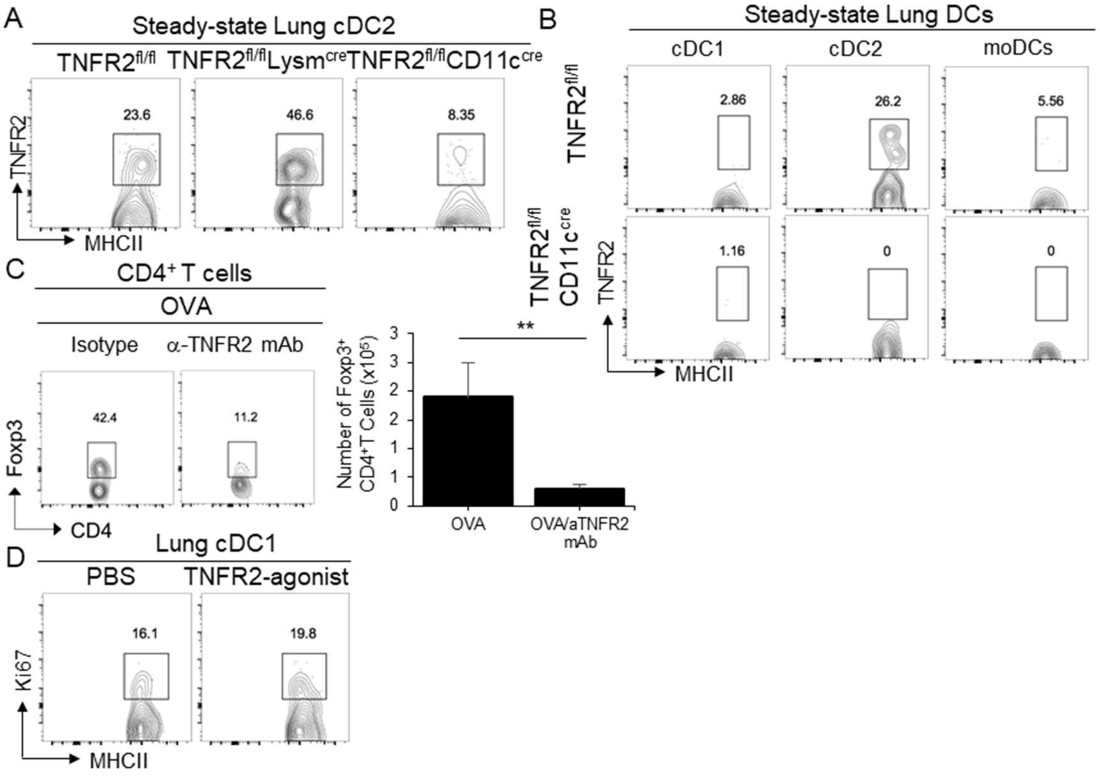

Supplemental Figure 3. Tonic TNFR2 signaling is required for the maintenance of R2D2. A. Flow cytometry analysis of lung TNFR2^{+} cDC2 at steady-state in TNFR2$^{fl/fl}$, TNFR2$^{fl/fl}$CD11c^{cre} and TNFR2$^{fl/fl}$Lysm^{cre} mice. **B.** Flow cytometry analysis of TNFR2 expression on lung DCs in TNFR2$^{fl/fl}$ and TNFR2$^{fl/fl}$CD11c^{cre} mice at steady-state. **C.** Flow cytometry analysis of T-regs in mice treated *i.n.* with OVA (1µg) and isotype antibody (20µg) or anti-TNFR2 blocking antibody (20µg). Lungs were harvested on day 14. n=3 mice/group. Data are representative of two independent experiments. **D.** Flow cytometry analysis of Ki67 expression in lung cDC1 from mice treated *i.n.* with anti-TNFR2 mAb (TR75.89) and TNFR2-agonist $TNF_{D221N/A223R}$ (1µg). Lungs were harvested 24 hours later. n=3 mice/group. Data are representative of two independent experiments. Graphs represent the mean with error bars indication s.e.m. P values determined by unpaired student t-test (C). *P<0.05, **P<0.001, ***P<0.0001.

Figure S4: IFNβ-IFNAR1 signaling in iR2D2 cells promotes T-regs induction in the lung

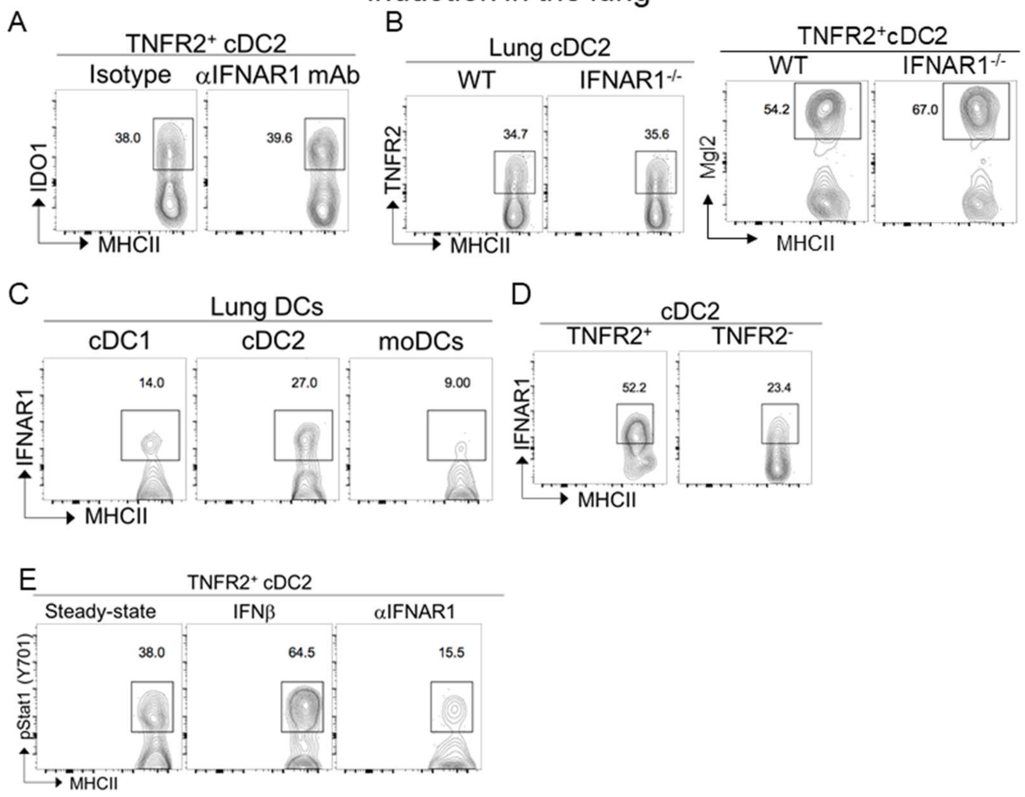

Supplemental Figure 4. IFNβ-IFNAR1 signaling in iR2D2 cells promotes lung T-regs induction. A. Flow cytometry analysis of IDO1 expression in TNFR2^{+} cDC2 in mice treated *i.n.* with isotype (20μg) or anti-IFNAR1 blocking antibody (20μg). Lungs were harvested 24 hours later. n=3mice/group. Data are representative of two independent experiments. **B.** Flow cytometry analysis of TNFR2^{+} cDC2 (left) and Mgl2^{+}TNFR2^{+} cDC2 (right) in WT and IFNAR1$^{-/-}$ mice at steady-state. n=3mice/group. Data are representative of three independent experiments. **C-D.** Flow cytometry analysis of IFNAR1 expression in lung DCs. n=3mice/group. Data are representative of two independent experiments. **E.** Flow cytometry analysis of pSTAT1 (Y701) (clone 58D6) expression in TNFR2^{+} cDC2 in mice treated *i.n.* with IFNβ (200ng), anti-IFNAR1 blocking antibody (20μg). Lungs were harvested 24 hours later. n=3mice/group. Data are representative of three independent experiments.

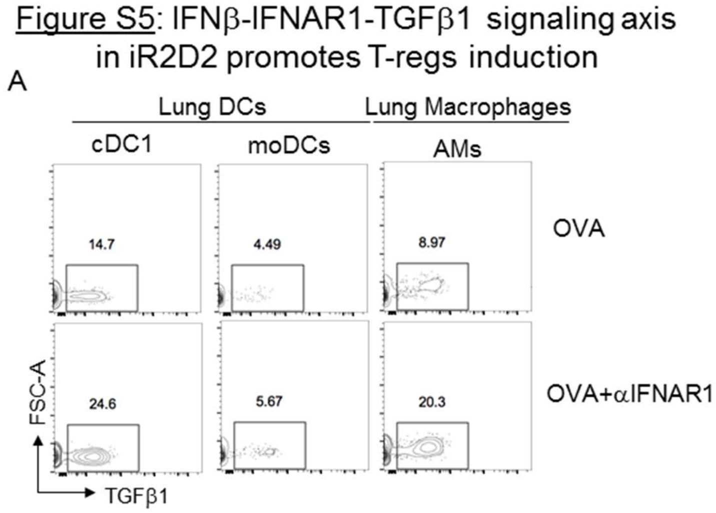

Supplemental Figure 5. IFNβ-IFNAR1-TGFβ1 signaling axis in iR2D2 promotes lung T-regs induction. A. Flow cytometry analysis of TGFβ1 expression in lung DCs and alveolar macrophages in WT mice treated with OVA (1μg) or anti-IFNAR1 blocking antibody (20μg). Lungs were harvested 24 hours later. n=3mice/group. Data are representative of two independent experiments.

Figure S6: R2D2 promotes T_H2 responses in HDM mice

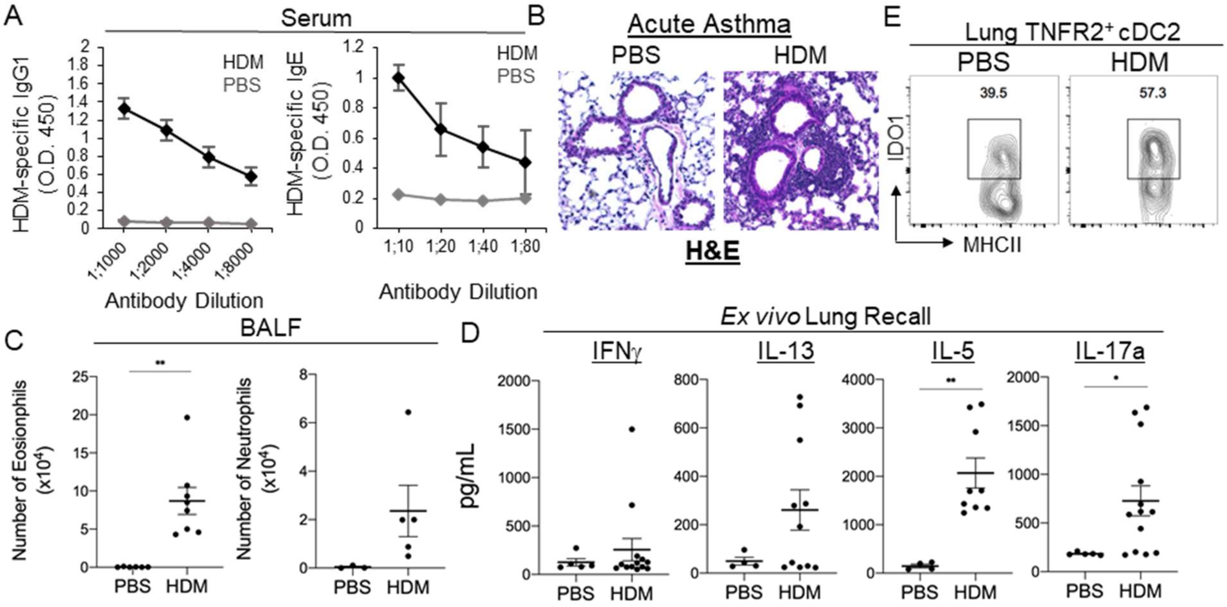

Supplemental Figure 6. iR2D2 promotes TH2 responses in HDM mice. A. Serum levels of HDM-specific IgG1 (left) and IgE from HDM-induced asthmatic mice. n=3 mice/group. Data are representative of four independent experiments. **B.** Representative haematoxylin and eosin (H&E) staining of lung sections of HDM-induced asthmatic mice. n=3mice/group. Data are representative of four independent experiments. **C.** Number of eosinophils (left) and neutrophils (right) in the BALF of HDM-induced asthmatic mice. Data were compiled two independent experiments. **D.** Lung samples from HDM-induced asthmatic mice were restimulated *ex-vivo* for 4 days with HDM (25μg/ml). Cytokines were measured using ELISA. Data were compiled from two independent experiments. **E.** Flow cytometry analysis of IDO1-expression on TNFR2$^+$ cDC2 in HDM-induced asthmatic WT mice. n=3 mice/group. Data are representative of three independent experiment. Graphs represent the mean with error bars indication s.e.m. P values determined by unpaired student t-test (C). *P<0.05, **P<0.001, ***P<0.0001.

Figure S7: Gating strategy for human lung dendritic cells

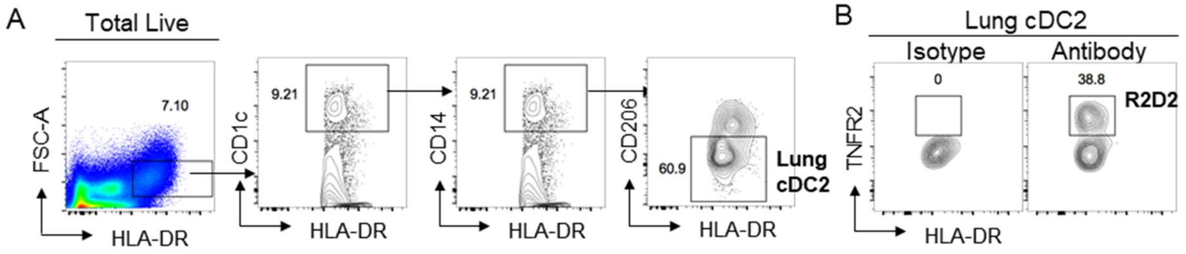

Supplemental Figure 7. Gating strategy for human lung dendritic cells. A. Human lung cDC2 are HLA-DR$^+$CD1c$^+$CD14$^-$CD206$^-$. **B.** Flow cytometry analysis of TNFR2$^+$ cDC2 in healthy human lung.
